# Targeting the Sugary Armor of *Klebsiella* Species

**DOI:** 10.3389/fcimb.2019.00367

**Published:** 2019-11-08

**Authors:** L. Ponoop Prasad Patro, Thenmalarchelvi Rathinavelan

**Affiliations:** Department of Biotechnology, Indian Institute of Technology Hyderabad, Kandi, India

**Keywords:** *Klebsiella* species, multidrug resistance, lipopolysaccharide, capsular polysaccharide, exopolysaccharide, complement system, vaccine, antibiotics

## Abstract

The emergence of multidrug-resistant strains of Gram-negative *Klebsiella* species is an urgent global threat. The World Health Organization has listed *Klebsiella pneumoniae* as one of the global priority pathogens in critical need of next-generation antibiotics. Compared to other Gram-negative pathogens, *K. pneumoniae* accumulates a greater diversity of antimicrobial-resistant genes at a higher frequency. The evolution of a hypervirulent phenotype of *K. pneumoniae* is yet another concern. It has a broad ecological distribution affecting humans, agricultural animals, plants, and aquatic animals. Extracellular polysaccharides of *Klebsiella*, such as lipopolysaccharides, capsular polysaccharides, and exopolysaccharides, play crucial roles in conferring resistance against the host immune response, as well as in colonization, surface adhesion, and for protection against antibiotics and bacteriophages. These extracellular polysaccharides are major virulent determinants and are highly divergent with respect to their antigenic properties. Wzx/Wzy-, ABC-, and synthase-dependent proteinaceous nano-machineries are involved in the biosynthesis, transport, and cell surface expression of these sugar molecules. Although the proteins involved in the biosynthesis and surface expression of these sugar molecules represent potential drug targets, variation in the amino acid sequences of some of these proteins, in combination with diversity in their sugar composition, poses a major challenge to the design of a universal drug for *Klebsiella* infections. This review discusses the challenges in universal *Klebsiella* vaccine and drug development from the perspective of antigen sugar compositions and the proteins involved in extracellular antigen transport.

## Introduction

*Klebsiella* species (spp.) are rod-shaped and encapsulated Gram-negative bacteria in the Enterobacteriaceae family (Podschun and Ullmann, 1998; Kenneth and Ryan, 2003; Murray and Baron, 2007; Paczosa and Mecsas, 2016). Eleven species have been identified in the *Klebsiella* genus, namely, *Klebsiella pneumoniae* (*K. pneumoniae*) (subsp. pneumoniae, subsp. *ozaenae*, subsp. *rhinoscleromatis*), *Klebsiella oxytoca (K. oxytoca), Klebsiella ornithinolytica (K. ornithinolytica), Klebsiella planticola (K. planticola), Klebsiella terrigena (K. terrigena)* (Murray and Baron, 2007), *Klebsiella variicola (K. variicola)* (Rosenblueth et al., 2004) [subsp. *tropicalensis* (Rodrigues et al., 2019)], *Klebsiella granulomatis (K. granulomatis)* (Carter et al., 1999)*, Klebsiella aerogenes (K. aerogenes)* (Tindall et al., 2017), *Klebsiella africanensis (K. africanensis)* (Rodrigues et al., 2019), *Klebsiella grimontii (K. grimontii)* (Passet and Brisse, 2018), and *Klebsiella quasipneumoniae (K. quasipneumoniae)* (subsp. *quasipneumoniae* and subsp. *similipneumonia*e) (Brisse et al., 2014). *Klebsiella pneumoniae (K. pneumonia)* (Kp) are further classified into classical (cKp) and hypervirulent (hvKp) strains based on their phenotype and nature of pathogenicity (Shon et al., 2013; Russo et al., 2018). *Klebsiella* spp. are generally found in animal and human gut microbiota (Selden et al., 1971; Taur and Pamer, 2013; Bilinski et al., 2016; Paczosa and Mecsas, 2016). They colonize a wide range of hosts including plants and mammals (Bagley, 1985; Podschun and Ullmann, 1998; Podschun et al., 2001; Wyres and Holt, 2018) and can grow ubiquitously in water and soil (Bagley, 1985; Podschun and Ullmann, 1998; Podschun et al., 2001; Rock et al., 2014).

*Klebsiella* spp. are generally opportunistic pathogens (Wyres and Holt, 2018) and do not usually affect healthy individuals (Bagley, 1985; Centers For Disease Control Prevention, 2010). Generally, it is immunocompromised individuals, such as patients undergoing chemotherapy, neonates, and the elderly, that are affected by cKp infections. In contrast, hvKp can infect healthy individuals of any age and can infect nearly every site of the body and spread metastatically (Liu et al., 1986; Fang et al., 2007; Russo et al., 2018). *Klebsiella* spp. utilize the following virulence traits to protect themselves from the host immune response (Davies, 2003; Lavender et al., 2004; Mishra et al., 2015; Paczosa and Mecsas, 2016; Hsieh et al., 2019): capsular polysaccharides (CPS), lipopolysaccharides (LPS), siderophores, fimbriae (alternatively, pili), a type VI secretion system, outer-membrane proteins, porins, efflux pumps, an iron transport system, biofilms, and allantoin metabolism. Among these, CPS, LPS, siderophores, and fimbriae are well-characterized virulence factors of *Klebsiella* spp. (Paczosa and Mecsas, 2016). These virulence factors assist *Klebsiella* spp. in evading the innate immune response of the host and to survive in different sites within the host, rather than actively suppressing host immune system components (Domenico et al., 1994; Hsieh et al., 2019). Notably, increased production of CPS and aerobactin (an iron-chelating siderophore) is specific to the hvKp pathotype (Cheng et al., 2010; Russo et al., 2018), as increased production of CPS results in a hypermucoviscous phenotype that has a viscous string length >5 mm (Cheng et al., 2010). Nevertheless, hypermucoviscosity is not specific to the hvKp pathotype, as cKp can also exhibit such a phenotype (Catalan-Najera et al., 2017; Russo et al., 2018). Furthermore, hvKp strains are not always hypermucoviscous (Catalan-Najera et al., 2017; Russo et al., 2018). Thus, the genes involved in the regulation of CPS (Cheng et al., 2010) and aerobactin production are used to distinguish the cKp and hvKp pathotypes (Russo et al., 2018). These are not elaborated here, as it is beyond the scope of this review.

*Klebsiella* spp. cause a variety of opportunistic nosocomial and community-acquired infections (Podschun and Ullmann, 1998; Tsai et al., 2008; Lin et al., 2010; Paczosa and Mecsas, 2016; Martin and Bachman, 2018; Vading et al., 2018; Juan et al., 2019), such as urinary tract infection (Goldstein et al., 1978; Sewify et al., 2016), soft tissue infection (Goldstein et al., 1978), pneumonia (Lee et al., 1996; Tan et al., 1998), septicemia (Arredondo-Garcia et al., 1992; Al-Anazi et al., 2008), bacteremia (Goldstein et al., 1978; Lin et al., 1997), meningitis (Price and Sleigh, 1972; Ku et al., 2017; Khaertynov et al., 2018), and pyogenic liver abscesses (Chowdhury and Stein, 1992; Youssef et al., 2012). As *Klebsiella* spp. have acquired resistance against various antimicrobials, they often become a challenge in treating these infections (Bengoechea and Sa Pessoa, 2019). For instance, Kp isolates have continuously accumulated resistance against four important classes of antibiotics, namely, the third-generation cephalosporins, aminoglycosides, fluoroquinolones, and carbapenems (Navon-Venezia et al., 2017; The European Antimicrobial Resistance Surveillance Network, 2018). Multiple drug resistance such as this eventually leads to extremely drug-resistant *Klebsiella* strains (XDR) (Magiorakos et al., 2012; Navon-Venezia et al., 2017).

In general, Kp is a hospital-associated pathogen that is subjected to continuous selective pressure due to continuous exposure to multiple antibiotics. *K. pneumoniae* inactivates a spectrum of beta-lactams through the action of carbapenemases and an extended spectrum of beta-lactamases (ESBL). As a consequence, Kp can become resistant to beta-lactams and thrive in healthcare settings (Hawkey and Jones, 2009; D'andrea et al., 2013; Andrade et al., 2014; Zhang et al., 2016; Feng et al., 2018; Fu et al., 2018). For example, a New Delhi metallo-β-lactamase 1 (NDM-1)-producing Kp strain originating from India has now disseminated across the globe (Yong et al., 2009; Khan et al., 2017). In 2016, a patient infected with NDM-1-producing Kp died due to a lack of treatment options in Nevada (Chen et al., 2017). Colistin, a drug of last resort that has been used against carbapenem-resistant *Enterobacteriaceae*, targets bacterial lipid A. *K. pneumoniae* has developed resistance against colistin through mutations in lipid A modification regulatory genes such as *mgrB* (Cannatelli et al., 2013; Jayol et al., 2014; Olaitan et al., 2014; Poirel et al., 2015; Wright et al., 2015). Although both cKp and hvKp are global pathogens, the former is predominantly found in Western countries, while the latter is observed in the Asia-Pacific Rim (Fazili et al., 2016; Rossi et al., 2018; Russo and Marr, 2019). However, the evolution of hvKp strains with multiple drug resistance (MDR) and extreme drug resistance (XDR) is due to either hvKp acquiring drug-resistant plasmids from cKp (Zhang et al., 2015, 2016; Wei et al., 2016; Feng et al., 2018; Fu et al., 2018; Yao et al., 2018) or cKp acquiring an hvKp virulence plasmid (Gu et al., 2018). Both pose a significant challenge with respect to the treatment of infection.

*Klebsiella pneumoniae* has evolved several mechanisms to resist antibiotics. In comparison to *Escherichia coli (E. coli)*, Kp has acquired double the number (more than 400) of antimicrobial-resistant (AMR) genes (Wyres and Holt, 2018). Interestingly, ESBL-producing Kp exhibits carbapenem resistance as a result of alterations in permeability due to loss of porins (Bradford et al., 1997; Martinez-Martinez, 2008; Leavitt et al., 2009) and overexpression of efflux pumps (Van De Klundert et al., 1988). *Klebsiella pneumoniae* has also acquired AMR through horizontal gene transfer enabled by plasmids and a mobile genetic environment (Pendleton et al., 2013). The emergence of plasmids with ESBL genes in Kp is one such example (Wachino et al., 2004; Queenan and Bush, 2007; Woodford et al., 2011; Lee et al., 2016). The translocation of carbapenemase-encoding genes from Kp plasmids onto a chromosome makes infections almost impossible to control (Lee et al., 2016). Due to chromosomal mutations, Kp has also become resistant to the antimicrobial peptide colistin (Olaitan et al., 2014; Doorduijn et al., 2016; Liu et al., 2016), leaving very few therapeutic options for the treatment of patients infected with Kp. Thus, it has become increasingly challenging to treat Kp infections, as reflected by the increase in the number of severe infections and the scarcity of effective antimicrobials (Paczosa and Mecsas, 2016).

As *Klebsiella* spp. are reservoirs for antibiotic-resistant genes (Navon-Venezia et al., 2017; Bengoechea and Sa Pessoa, 2019), they can act as key traffickers of AMR genes to other environmentally and clinically important Gram-negative bacteria. One such example is spread of carbapenem resistance genes from Kp (Sidjabat et al., 2009) strains originated in the United States (Smith Moland et al., 2003) to other Gram-negative bacterial species such as *Salmonella* (Miriagou et al., 2003), *Enterobacter* spp. (Hossain et al., 2004), *Escherichia coli* (Bratu et al., 2007), and *Proteus mirabilis* (Tibbetts et al., 2008). Such examples of interspecies spread have been observed for quite some time. Similar to Kp, other *Klebsiella* species have also acquired resistance against antibiotics. *Klebsiella grimontii* (which is closely related to *Klebsiella oxytoca*) is a newly added species to the *Klebsiella* genus (Passet and Brisse, 2018) and has acquired resistance against carbapenem (Liu et al., 2018). These events prompted the World Health Organization (WHO) to call for a global effort to develop next-generation antibiotics against *Klebsiella* infections (World Health Organization, 2017, 2018).

The rise in multidrug-resistant *Klebsiella* spp. (as well as hvKp strains) and their periodic outbreak and global spread (Navon-Venezia et al., 2017) warrant a new treatment strategy, along with a new set of antibiotics and vaccines for *Klebsiella* infections. Targeting bacterial survival mechanisms (rather than destroying the bacteria) exerts less selective pressure on the bacteria. For example, targeting the sugary armor of *Klebsiella* spp., such as the LPS, CPS, and exopolysaccharide (EPS), would be an efficient alternative strategy. Though structural information and mechanical insights relating to the transport of CPS and LPS onto the bacterial surface through various proteinaceous nanomachines are available (Rahn et al., 1999, 2003; Kos et al., 2009; Ruiz et al., 2009; Shu et al., 2009; Freinkman et al., 2012; Sachdeva et al., 2017; Bi et al., 2018), only a fragmented picture of their utility as potential drug and vaccine targets exists. To this end, this review focuses on targeting the CPS, LPS, and EPS armors of *Klebsiella* spp.

## Host Innate Immune Defenses Against *Klebsiella* Species

When a pathogen enters a host, it must contend with the mechanical, chemical, and cellular barriers exhibited by the host, and *Klebsiella* spp. is no exception (Zhang et al., 2000). Initially, it has to overcome mechanical barriers such as the epithelia of the skin, mucociliary clearance, the low-pH environment of the genitourinary tract or gastrointestinal tract, etc. Subsequent to this, the pathogen must circumvent the humoral and cellular innate defenses. Several humoral defenses (opsonic, bactericidal, and bacteriostatic) are used by the host for bacterial clearance (Kabha et al., 1997; Zhang et al., 2000; Ivin et al., 2017). One such humoral defense is the complement system, which is activated in three different pathways (namely, the classical, alternative, and mannose-binding lectin pathways) (Murphy et al., 2012) for the purpose of clearing bacteria. In addition, the pathogen has to deceive antimicrobial peptides, collectins, and cellular components (i.e., neutrophils, monocytes/macrophages, dendritic cells, and innate lymphoid cells) of the innate immune defense to survive and maintain its growth in the host (Murphy et al., 2012). The mechanisms of *Klebsiella* spp. defense against the host are covered in detail in recent reviews (Doorduijn et al., 2016; Paczosa and Mecsas, 2016; Bengoechea and Sa Pessoa, 2019).

Once *Klebsiella* spp. overcome the mechanical barriers of the host, a variety of host immune defense pathways are activated by pathogen recognition receptors (PRRs) such as “Toll-like” receptors (TLRs), nucleotide-binding oligomerization domain-like receptors (NLRs), etc. (Takeuchi and Akira, 2010) through the detection of pathogen-associated molecular patterns (PAMPs). As CPS and LPS are major pathogen surface components, many of the PRRs activate these immune response pathways primarily mediated by the detection of LPS and CPS. For instance, upon binding to TLR4, the CPS activates the NF-κB-mediated inflammatory and immune response pathways (Regueiro et al., 2006, 2009; Yang et al., 2011). Interaction of LPS with TLR4 and MD2 receptors on the host innate immune cells also induces the NF-κB-mediated inflammatory response (Kawai and Akira, 2010; Maeshima and Fernandez, 2013). The lung collectins SP-A and SP-D, which are soluble PRRs, bind to LPS and facilitate agglutination and phagocytosis by macrophages. The recruitment of the classical complement pathway (following LPS detection) and that of the lectin-mediated complement pathway (upon detection of CPS) are some of the major host strategies for bacterial clearance (Walport, 2001; Ricklin et al., 2010; Holers, 2014; Gomez-Simmonds and Uhlemann, 2017). On detection of LPS, NLR protein family members assemble to form inflammasome, which activates caspase 4/5 in humans and caspase-11 in the mouse. This triggers the activation of non-canonical inflammasome to produce IL1β and induce bacterial cell death (Hagar et al., 2013; Shi et al., 2014). Opsonophagocytosis mediated by neutrophils and macrophages is also a major bacterial clearance strategy (Domenico et al., 1994; Salo et al., 1995; Regueiro et al., 2006).

## Host Immune Evasion Strategies of *klebsiella* spp.

*Klebsiella* spp. make use of several sophisticated stealth immune evasion strategies to escape from the host innate immune response, rather than actively suppressing it. However, recent research indicates that *Klebsiella* spp. have also developed several anti-immune strategies that involve the attack of key regulators and effectors of the host immune system. This makes them formidable pathogens capable of disseminating and growing across a variety of sites in their hosts (Paczosa and Mecsas, 2016; Bengoechea and Sa Pessoa, 2019). To establish in the host, the pathogen has to counteract the host innate immune defenses (Zhang et al., 2000). The surface oligosaccharide molecules (CPS and LPS) are some of the major virulence factors that *Klebsiella* spp. use to protect themselves from the host immune response.

### Capsular Polysaccharide

The surface of *Klebsiella* spp. is shielded by a thick layer of CPS fibers that protect the bacteria from the environment (Amako et al., 1988). The polysaccharide capsule assists the bacteria in surviving stressful environmental conditions such as desiccation and exposure to detergents. High-molecular-weight CPS, consisting of linear or branched oligosaccharides, form a shield around the *Klebsiella* spp. cell surface and represent a physical barrier against the complement system, as also seen in *E. coli* (Meri and Pangburn, 1994; Alvarez et al., 2000; Cortes et al., 2002b; Abreu and Barbosa, 2017). This shield plays a crucial role in protecting Kp against innate immune response mechanisms, evading complement deposition and opsonization, reducing recognition, and adhesion by epithelial cells and phagocytes, and abrogating lysis by antimicrobial peptides and complement cascades (Podschun and Ullmann, 1998; Fang et al., 2004; Lin et al., 2004; Pomakova et al., 2012; Paczosa and Mecsas, 2016; Martin and Bachman, 2018). Poorly encapsulated Kp strains are readily vulnerable to phagocytosis (Cortes et al., 2002a; De Astorza et al., 2004). As compared to capsular Kp strains, acapsular Kp strains are more easily phagocytosed by innate immune cells (Domenico et al., 1994; Yoshida et al., 2000; Cortes et al., 2002b; Lawlor et al., 2005, 2006). Deletion of the genes responsible for capsule formation in the clinical strains ideally leads to a non-pathogenic bacterium by drastically impairing the virulence of Kp (Cortes et al., 2002b; Lawlor et al., 2006). It has been shown that the thickness of CPS (rather than its chemical composition) determines the extent of protection it confers to *Klebsiella* spp. (De Astorza et al., 2004). Not surprisingly, hvKp exhibits enhanced resistance to a variety of humoral defenses such as complement killing, HBD-1 to HBD-3 [human beta-defensin (HBD)], and to antimicrobial peptides such as neutrophil protein 1 and lactoferrin (Fang et al., 2004).

The capsule type, also known as the K-antigen or K-type, is *Klebsiella* species*-*specific and is widely used in the serotyping of *Klebsiella* spp. Traditionally, *Klebsiella* spp. are identified as having 77 K-antigens (viz., K1–K81, excluding K75–K78) based on the diversity in their sugar composition, type of glycosidic linkage, and the nature of enantiomeric and epimeric forms (https://iith.ac.in/K-PAM/, K-PAM unpublished) (Pan et al., 2015). Recently, additional K-types have been identified based on the CPS locus or K-locus (KL) arrangement. These are known as the KL series (KL1–KL81, KL101–KL149, KL151, KL153–KL155, and KL157–159) (Wyres et al., 2016). It is noteworthy that the KL1–KL81 locus types and the K1–K81 K-types are synonymously used. However, the sugar compositions of the remaining antigens in the KL series are as yet unknown. The variation in the repeating units of different K-antigens leads to varying degrees of detection of *Klebsiella* spp. by the innate immune system (Kabha et al., 1995; Doorduijn et al., 2016). Among the 134 K-types (including the KL series) identified so far (Wyres et al., 2016), only a few of them are frequently found in the strains isolated from clinical samples (Cryz et al., 1986). Due to the increased production of CPS, hypervirulent Kp strains produce a hypercapsule, which is a hypermucoviscous EPS bacterial coating that may significantly contribute to Kp pathogenicity (Shon et al., 2013). *Klebsiella pneumoniae* strains with a hypercapsule are less sensitive to complement detection and elimination (Pomakova et al., 2012) and also have increased resistance to phagocytosis (Fang et al., 2004; Lin et al., 2004; Pomakova et al., 2012) compared to the classical strains. However, some cKp strains are also found to have a hypermucoviscous coating (Catalan-Najera et al., 2017; Russo et al., 2018). Notably, the presence of fucose in the hypercapsule has been implicated in the evasion of the immune response for the K1 antigen (Wu et al., 2008; Yeh et al., 2010). Although Kp strains possessing K1 and K2 serotypes are often found to be hypervirulent (Fung et al., 2002; Struve et al., 2015), other capsule types such as K5, K20, K47, K54, K57, and K64 are also found in hvKp strains (Yu et al., 2008; Shon et al., 2013; Russo et al., 2018).

*Klebsiella* spp. K-antigens are negatively charged (as is the case for other Gram-negative bacteria) and consist of up to six monosaccharides in their main chain as well as in the branch: D-mannose, D-glucose, D-galactose, L-fucose, and L-rhamnose. Of particular note is a completely new monosaccharide, 4-deoxy-threo-hex-4-enopyranosyluronic acid, that is found in K38 but is absent in any other K-antigen structure (Jansson et al., 1994). Detailed analyses of *Klebsiella* spp. K-antigen sugar compositions (Table 1) reveal that as with *E. coli* (Kunduru et al., 2016), K-antigens are negatively charged due to the presence of uronic acid and or pyruvate substitutions (https://iith.ac.in/K-PAM/, unpublished work). Additionally, they also have O-acetyl, O-lactose, O-formyl, and glutamate substitutions. The evolution and variability in the sugar composition of CPS are one of the major advantages possessed by *Klebsiella* when evading the host immune response.

**Table 1:** Main-chain and side-chain sugar composition of 79 K-antigens of *Klebsiella* spp.

	**Main chain sugars**																											**side chain sugars**																				
**K-antigen**	**α-L-Fuc*p***	**α-L-Fuc-Oac**	**β-D-Gal*f***	**α-D-Gal*p***	**α-D-Gal*p*-pyr**	**α-D-Gal*p*A**	**β-D-Gal*p***	**β-D-Gal*p*-Oac**	**β-D-Gal*p*-pyr**	**α-D-Glc*p***	**α-D-Glc*p*A**	**β-D-Glc*p***	**β-D-Glc*p*-Oac**	**β-D-Glc*p*-pyr**	**β-D-Glc*p*A**	**β-D-Glc*p*A-pyr**	**β-D-Glc*p*A-Oac**	**α-D-Man*p***	**α-D-Man*p*-Oac**	**β-D-Man*p***	**β-D-Man*p*-Oac**	**β-D-Man*p*-pyr**	**α-L-Rha*p***	**α-L-Rha*p*-OAc**	**α-L-Rha*p*-pyr**	**β-L-Rha*p***	**K-antigen**	**α-D-Gal*p***	**α-D-Gal*p*-pyr**	**α-D-Gal*p*A**	**β-D-Gal*p***	**β-D-Gal*p*-pyr**	**α-D-Glc*p***	**α-D-Glc*p*A**	**β-D-Glc*p***	**β-D-Glc*p*-pyr**	**β-D-Glc*p*-Olac**	**β-D-Glc*p*-Ofor**	**β-D-Glc*p*A**	**β-D-Glc*p*A-Glu**	**β-D-Glc*p*A-Olac**	**α-D-Man*p***	**α-D-Man*p*-pyr**	**β-D-Man*p***	**β-D-Man*p*-pyr**	**α-L-Rha*p***	**β-L-Rha*p*-pyr**	**Extra**
K1	1											1				1											K1																					
K2										1		1								1							K2							1														
K3						1	1											2									K3																1					
K4										1	1	1						1									K4																					
K5													1		1							1					K5																					
K6	1										1	1										1					K6																					
K7												1		1	1			2									K7	1																				
K8				1			1		1			1															K8												1									
K9				1																			3				K9												1									
K10				1			1			1		1			1			1									K10	1																				
K11				1								1			1												K11		1																			
K12			1	1						1													1				K12					1							1									
K13										1		1								1							K13					1		1														
K14			1									1			1					1							K14									1										1		
K15							4																				K15							1	1													
K16	1									1					1												K16				1																	
K17											1	1											1			1	K17																			1		
K18							1			1													1				K18						1						1							1		
K19							1			1	1												2				K19																			1		
K20							1											1									K20	1											1									
K21a							1				1							2									K21a		1																			
K21b							1										1	2									K21b		1																			
K22								1				1															K22						1								1							
K23												1											1				K23						1						1									
K24										1					1			2									K24																	1				
K25							1					1															K25								1				1									
K26							1				1							2									K26					1	1		1													
K27							−2					1		1													K27								1				1									
K28				1								1						2									K28								1				1									
K30												1								2							K30					1		1														
K31							1				1	1															K31									1						1						
K32				1																			1		1	1	K32																					
K33												1								1	1						K33					1		1														
K34						1						1											3				K34																			1		
K35					1							1						2									K35												1									
K36							1																3				K36									1			1									
K37							1					1															K37						1								1							
K38				1			1					1															K38								1													β-L-Sug
K39												2			2			2									K39																					
K40				1							1							2					2				K40																					
K41			1	1						1													1				K41						1		1				1									
K43				1														2									K43												1					1				
K44										1		1			1								2				K44																					
K45												1											3				K45												1									
K46				1			1				1							1									K46								1										1			
K47							1																1				K47												1							1		
K48										1		1											2				K48			1																		
K49				2														1									K49			1																		
K50							1			1	1							2									K50				1		1															
K51				2																							K51						1	1														
K52							−3								−1								−1				K52																			−1		
K53							1								1			2					1				K53																			1		
K54	1	1									2	2															K54											2										
K55												1												1			K55	1						1														
K56				1			2							1													K56																			1		
K57						1	1											1									K57															1						
K58		1								1						1											K58	1																				
K59							1					1							2								K59												1									
K60				1								1			1			1									K60						1		2													
K61										1		1			1			1									K61	1																				
K62							1			1					1			1									K62															1						
K63	1			1		1																					K63																					
K64											1	1						2									K64									1										1		
K66				1							1							2									K66										1											
K67												1						2					1				K67				1								1							1		
K68						1	1											1									K68																1					
K69												1								2							K69					1		1														
K70							1			1					1								2		1		K70																					
K71												1											3				K71								1				1							1		
K72												1											2		1		K72																					
K73							1					1														1	K73												1									
K74							1											2									K74					1		1														
K79							1								1								3				K79						2															
K80							1											2									K80							1													1	
K81							1								1								4				K81																					
K82				1				1				1															K82													1								
K83							1																1				K83	1						1														

*Note that the number of occurrences of a particular sugar (which varies between 1 and 4) is also indicated. The presence of substitution(s) is also indicated next to the corresponding sugar. The sugar compositions of the newly identified CPS locus [KL series (Wyres et al., 2016)] are not given, as they are unknown. The K-antigen names highlighted in the purple cells employ WbaP as the initiating glycosyl-transferase, while the K-antigens in the white cells use WcaJ protein as the initiating glycosyl-transferase. The number of sugars in CPS structures with unknown anomeric forms is represented by negative values. Note that following abbreviations are used for sugar molecules in the table*.

*Sugar name: α-L-Fucp*.

*Position: 1-2-3456*.

*1: α and β represent the anomeric forms of the sugar molecules*.

*2: D and L represent the enantiomers of the sugar molecule*.

*345: Tri-letter sugar code (see below)*.

*6: The molecule name terminates with “p” or “f” is for pyranose or furanose sugar forms respectively*.

*Fuc, Fucose; Gal, Galactose; Glc, Glucose; Rha, Rhamnose; Man, Mannose; GalpA, Galacturonic acid (Pyranose); GlcpA, Glucoronic acid (Pyranose); Oac, O-acetyl group; Pyr, pyruvyl group; β-L-Sug, 4-deoxy-three-hex-4-enopyranosyluronic acid*.

### Lipopolysaccharide

Pathogenicity factor LPS, also known as endotoxin, is found on the bacterial outer leaflet of the outer membrane and plays an important role in offering protection against cationic antimicrobial peptides (Clements et al., 2007; Llobet et al., 2015) and the complement system in certain serotypes (Merino et al., 1992). *Klebsiella pneumoniae* exploits the versatility of both CPS and LPS to counteract the complement system (Ciurana and Tomas, 1987; Alvarez et al., 2000; Shankar-Sinha et al., 2004; Doorduijn et al., 2016; Adamo and Margarit, 2018). It has been shown that purified LPS from Kp inhibits serum-mediated clearance (Merino et al., 1992). The structure of LPS consists of lipid A, core oligosaccharides, and O-antigens, among which the O-antigen composition is highly variable across different strains of *Klebsiella* spp. (Table 2, http://iith.ac.in/K-PAM/o_antigen.html) (Lugo et al., 2007). Unlike K-antigens, *Klebsiella* spp. has only 11 O-antigens (Clarke et al., 2018). O-antigen is also used in the typing of *Klebsiella* spp. Among the 11 O-antigen types found in *Klebsiella* spp., O1, O2, O3, and O5 are found in clinically imported strains (Hansen et al., 1999; Follador et al., 2016). O-antigens of *Klebsiella* spp. consist of D-galactose, D-galactofuranose, D-mannose, D-ribofuranose, and N-acetyl-D-glucosamine sugars. Their composition varies between different O-antigens, leading to differences in their antigenicity. Like the K-antigen, the O-antigens differ from each other in terms of sugar composition, glycosidic linkage, number of repeating units, and epimeric and enantiomeric forms (https://iith.ac.in/K-PAM/, unpublished work) (Follador et al., 2016; Clarke et al., 2018). Unlike the K-antigens, only acetyl group substitution is observed in O-antigens (and only in one of the O-antigens). While *Klebsiella* spp. strains that have truncated O-antigen or lack O-antigen (termed as “rough LPS”) are susceptible to complement system-mediated killing, the full-length O antigen or smooth LPS-containing *Klebsiella* spp. strains are resistant to complement system-mediated killing (Ciurana and Tomas, 1987; Mccallum et al., 1989; Merino et al., 1992). Although the complement-resistant strains activate the complement cascade, they are not susceptible to killing, as O-antigen variability protects the Kp surface molecules (Merino et al., 1992; Alberti et al., 1996; Shankar-Sinha et al., 2004; Merle et al., 2015).

**Table 2:** Main-chain and side-chain sugar compositions of 11 O-antigens of *Klebsiella* spp. Note that the number of occurrences of a particular sugar (which varies between 1 and 5) is also indicated.

	**Main chain sugars**									**side chain sugars**	
**O-antigens**	**β-D-Rib*f***	**β-D-Gal*f***	**β-D-Gal*f*-2OAc & 6O-Ac**	**α-D-Gal*p***	**β-D-Gal*p***	**β-D-GlcNAc**	**α-D-Man*p***	**β-D-Man*p***	**α-L-Rha*p***	**O-antigens**	**α-D-Gal*p***
O1				1	1					O1	
O2a		1		1						O2a	
O2c		1				1				O2c	
O2aeh		1		1						O2aeh	1
O2afg		1		1						O2afh	1
O3							5			O3	
O4	1			1						O4	
O5							2	1		O5	
O7	1								3	O7	
O8			1	1						O8	
O12						1			1	O12	

*The presence of substitution(s) is also indicated next to the corresponding sugar (Clarke et al., 2018). The abbreviations used for sugar molecules are as per Table 1. The sugars mentioned for the first time (in this table) are: GlcNAc, N Acetyl Glucosamine; Ribf, Ribofuranose*.

### Exopolysaccharide

The extracellular matrix (which is a component of bacterial biofilm) of *Klebsiella* spp. is composed of proteinaceous adhesins, nucleic acids, and EPS (Sutherland, 2001; Branda et al., 2005; Vu et al., 2009). Compared to other surface-attached polysaccharides, little information is available on biofilm-associated EPS, which is yet another virulence factor of Kp (Cescutti et al., 2016). It has been shown that biofilm polysaccharides of Kp to some extent reduce antimicrobial peptide activity by preventing it from reaching the bacterial membrane or by impeding interaction with the membrane (Bellich et al., 2018). Genetic information regarding the biosynthesis of EPS is encoded in specific operons on the bacterial genome and 30 ORFs have been identified for the hetero-capsular EPS K40-type of *Klebsiella* spp. (Pan et al., 2015). In general, EPS contains rare sugars such as l-fucose, l-rhamnose, or uronic acids (Kumar et al., 2007), and *Klebsiella* is no exception. For example, hexasaccharide repeats of *Klebsiella* I-714 EPS have a high l-rhamnose content in addition to d-galactose and d-glucuronic acid (López-Santin, 1995; Roca et al., 2015). The primary structures of EPS extracted from *K. pneumoniae* strain KpTs113 have K24 CPS-repeating units and the *K. pneumoniae* strain KpTs101 is identical to the O1 antigen of LPS. This observation is supported by the finding that CPS and LPS are required for building the mature biofilm architecture (Balestrino et al., 2008; Benincasa et al., 2016; Cescutti et al., 2016). However, the KpMn7 strain has a rare sugar (rhamnose) in the repeating unit and is highly similar (but not identical) to the K24 CPS unit. Given this intriguing finding, further research is warranted on novel EPS structures found in *Klebsiella* spp. (Kubler-Kielb et al., 2013; Bellich et al., 2018).

## Extracellular Polysaccharide Biosynthesis and Transportation Pathways

The aforementioned extracellular polysaccharide virulence factors are biosynthesized in the cytoplasm and transported through sophisticated proteinaceous nano-machines onto the bacterial surface. In general, bacteria use three different pathways for the transport of extracellular polysaccharides: (i) a Wzx/Wzy-dependent pathway (Rahn et al., 1999; Whitfield, 2006; Kalynych et al., 2014), (ii) an adenosine tri-phosphate (ATP)-binding cassette (ABC) transporter-dependent pathway (Cuthbertson et al., 2010; Greenfield and Whitfield, 2012; Kalynych et al., 2014), and (iii) a synthase-dependent pathway (Whitney and Howell, 2013). In addition to these, a fourth pathway (the dextrase/sucrase-dependent pathway) has also been identified for EPS secretion (Whitney and Howell, 2013; Schmid et al., 2015; Schmid, 2018).

*Klebsiella* spp. use a Wzx/Wzy-dependent pathway for CPS secretion (Rahn et al., 1999) that is similar to Group 1 CPS surface export in *E. coli* (Rahn et al., 1999; Whitfield and Paiment, 2003; Sachdeva et al., 2017). *Klebsiella* spp. use three independent ABC transporter-dependent pathways for LPS secretion. Although it is known that *E. coli* uses a Wzx/Wzy-dependent pathway for EPS secretion (Reid and Whitfield, 2005), there is no information available regarding EPS secretion in *Klebsiella* spp. Understanding the mechanisms of transport and the structural features of the proteins involved in such transport is essential for the identification of potential antimicrobial targets and the development of novel antimicrobials. As information on the EPS-secretion pathway is not available for *Klebsiella* spp., the following sections are limited to a review of CPS and LPS transportation strategies used by *Klebsiella*. The structures of the proteins involved in *Klebsiella* CPS and LPS export (with the exceptions of LptDE and LptB_2_FG) have been obtained by homology modeling using known template structures from other organisms (see Table 3).

**Table 3:** Details of the protein structures used in Figures 1, 2.

**Components of CPS/LPS biosynthesis and transportation pathway**	**PDB ID (available)**	**Organism name for which crystal structure is available**	**Genbank ID of the Klebsiella sequences used for the homology modeling (% sequence identity with available PDB structure)**
**CPS BIOSYNTHESIS AND TRANSPORTATION PATHWAY (FIGURE 1)**			
Wzi	2YNK	*Escherichia coli*	BAF47011.1 (99.78%)
Wza	2J58	*Escherichia coli*	BAF47012.1 (99.44%)
Wzb	2WMY	*Escherichia coli*	BAF4703.1 (99.32%)
Wzc (cytoplasmic domain)	3LA6	*Escherichia coli* (strain K12)	BAF47029.1 (57.93%)
Wzx	3MKU	*Escherichia coli*	BAT24471.1 (14.11%)
**LPS BIOSYNTHESIS AND TRANSPORTATION PATHWAY (FIGURE 2)**			
Wzm	6AN7/6OIH	*Aquifex aeolicus* (strain VF5)	CZQ25306.1 (34.5%)
Wzt-NBD	6AN5	*Aquifex aeolicus* (strain VF5)	CZQ25307.1 (46.32%)
Wzt-CBD	2R5O	*Escherichia coli*	CZQ25307.1 (100%)
MsbA	3B60	*Salmonella typhimurium*	SSW84925.1 (94.7%)
LptB_2_FG	5L75	*K. pneumoniae*	
LptDE	5IV9	*K. pneumoniae*	

### The Wzx/Wzy-Dependent Secretion Pathway

The chromosomal *cps* gene cluster harbors genes that are essential for the biosynthesis of sugar precursor molecules, assembly of the repeating unit, flipping of the repeating unit to the periplasmic side, polymerization of the repeating unit, transport of the nascent CPS, and anchorage of CPS onto the surface of *Klebsiella* (Pan et al., 2015). *Klebsiella* spp. utilize a Wzx/Wzy-dependent CPS secretion pathway, which is similar to that for Group 1 capsule production in *E. coli* (Whitfield and Paiment, 2003; Sachdeva et al., 2017). The process of CPS export in *Klebsiella* spp. begins with the biosynthesis of nucleotide sugar precursors corresponding to a particular K-type and the assembly of the repeat unit at the cytoplasmic face. This occurs with the help of sugar-specific glycosyl transferases encoded by genes such as *wbaP, wcaN, manC, rmlA, wcaA, wcuD, wcuM, wckA*, and *wclH* (Rahn et al., 1999; Shu et al., 2009; Pan et al., 2015). Subsequently, recognition of the specific CPS-repeating unit by the flippase Wzx occurs with the first sugar linked to undecaprenol-pyrophosphate (Und-PP), followed by flipping to the periplasmic side. Repeat unit polymerization is facilitated by Wzy copolymerase (Whitfield and Paiment, 2003; Li et al., 2016). Finally, Wza (an outer-membrane translocon), Wzc (a tyrosin autokinase), and Wzb (a phosphatase) synergistically transport CPS onto the bacterial surface and anchor the CPS onto the outer-membrane protein Wzi (Rahn et al., 2003; Whitfield, 2006; Woodward et al., 2010). This CPS export pathway is common to all *Klebsiella* spp. (as they all have *cps* locus genes). Gene sequences of the *cps* locus (specifically *wzi* and *wzc*) are used in the K-typing of *Klebsiella* spp., owing to limitations in conventional K-typing (Brisse et al., 2013; Pan et al., 2013; Wyres et al., 2016). *Klebsiella* spp. *cps* gene sequences (e.g., *wzi, wza, wzb, wzc, wbap, wcaj, wzx*, and *wzy*) vary according to their K-antigen composition and are used in genome-based surveillance of *Klebsiella* spp. (Pan et al., 2015; Wyres et al., 2016) (https://iith.ac.in/K-PAM/, unpublished work). Notably, a recent study has shown that the arrangement of the genes in the CPS locus is K-type-specific and this finding has been successfully applied to *Klebsiella* spp. K-typing (Pan et al., 2015; Wyres et al., 2016; Wick et al., 2018). It has been found that a *Klebsiella* spp. strain can either contain initialization glycosyl transferase WbaP or WcaJ, but not both (Shu et al., 2009). Sugar composition analysis indicates that the serotypes K1, K2, K4-K8, K11, K13, K14, K16, K17, K22–K25, K28, K30, K31, K33–K35, K37, K39, K44, K45, K48, K54, K55, K58–K61, K64, K67, K69, K71–K73, and K82 have WcaJ and use glucose-Und-PP as an initializing sugar. On the other hand, K3, K9, K10, K12, K15, K18–K21, K26, K27, K32, K36, K38, K40, K41–K43, K46, K47, K49–K53, K56, K57, K62, K63, K66, K68, K70, K74, and K79–K81 use galactose-Und-PP as the initializing sugar and have WbaP in their *cps* gene cluster.

### LPS Biosynthesis in the Cytoplasm by ABC-Dependent Pathway

LPS are glycolipids that encompass three structural moieties, namely, lipid A, core oligosaccharides (core-OS), and the O-antigenic polysaccharide (O-PS) (Whitfield and Trent, 2014). The lipid A (the lipid moiety of LPS) is highly conserved and anchors the LPS on the outer leaflet of the outer bacterial membrane. The core-OS is conserved and acts as a linkage between the lipid A and O-PS. The O-PS is highly variable across different *Klebsiella* spp.

Such complexity in the LPS structure leads to a complex biosynthesis pathway that takes place at the cytosolic and periplasmic faces of the inner membrane: (i) biosynthesis of lipid A through the Raetz pathway, (ii) attachment of core-OS to the lipid A, (iii) flipping of the lipid A-core-OS to the periplasmic end, (iv) biosynthesis of O-PS at the cytoplasmic end, (v) flipping of O-PS to the periplasmic region, and (vi) ligation of O-PS to lipid A-core-OS in the periplasmic region (Raetz and Whitfield, 2002; Whitfield and Trent, 2014). Finally, the LPS molecule assembled in the periplasmic region is exported to the bacterial surface wherein lipid A acts as the anchorage point for the LPS (Okuda et al., 2016). The entire process of LPS biosynthesis and surface export involves four different gene clusters: *lpx, waa, rfb*, and *lpt*. The gene products of *lpx, waa*, and *rfb* are involved in the biosynthesis of lipid A, core-OS, and O-PS, respectively (Regue et al., 2005; Fresno et al., 2007; Okuda et al., 2016). The *lpt* gene products are involved in the transport of the LPS molecule to the extracellular side of *Klebsiella*. The other protein involved in this biosynthesis process is MsbA, which is part of a different gene cluster. Intriguingly, *Klebsiella* spp. LPS biosynthesis and transportation are driven by ATP hydrolysis at three different stages: flipping of the lipid A-core-OS, flipping of O-PS, and transport of LPS from the periplasmic end to the bacterial extracellular region. These steps are outlined below.

#### Biosynthesis of Kdo_2_-lipid A–core-OS

The biosynthesis of lipid A begins in the cytosolic region with the involvement of nine enzymes synthesized from the *lpx* gene cluster (Raetz et al., 2009). The first step is the substitution of an acyl chain to the 3-OH group of uridine diphosphate N-acetylglucosamine (UDP-GlcNAc) (Anderson et al., 1985; Anderson and Raetz, 1987), followed by the release of an acetate group and the addition of the second acyl side chain. Two such monosaccharides are glycosylated, wherein one is phosphorylated (called lipid X) prior to the reaction, following which disaccharide-1-phosphate is again phosphorylated to synthesize Lipid IV_A_ at the cytoplasmic face of the inner membrane. The matured lipid IV_A_ is glycosylated with two 3-deoxy-D-manno-oct-2-ulosonic acid (Kdo) residues, which are incorporated by WaaA (a product of the *waa* gene cluster) to produce Kdo_2_-lipid IV_A_. Subsequently, Kdo_2_-lipid A is synthesized by the acylation of Kdo_2_-lipid IV_A_. This entire Raetz pathway takes place in the cytoplasmic end of the inner membrane and is mediated by several glycosyltransferases, along with other enzymes (Raetz and Whitfield, 2002).

In the next step, core-OS is synthesized by extending Kdo_2_-lipid A with the help of several glycosyltransferase enzymes (which vary as per the sugar components of different O-antigens). In general, the core-OS is conceptually divided into two regions, namely, the conserved inner core and the variable outer core. The inner core typically has Kdo_2_ and L-glycero-D-manno-heptopyranose (L, D-Hep). The outer core consists of three to six sugars, whose compositions are variable.

#### Export of Kdo_2_-lipid A–core-OS Across the Inner Membrane

The nascent Kdo_2_-lipid A–core-OS intermediate is subsequently flipped to the periplasmic end of the inner membrane through an ABC transporter, MsbA. MsbA is a “half” transporter as it contains two different polypeptide chains wherein each chain contains a nucleotide-binding domain (NBD) and a transmembrane domain (TMD). MsbA uses an “outward only” mechanism to flip Kdo_2_-lipid A–core across the inner bacterial membrane. In this mechanism, MsbA remains in a resting state with an (inward) open conformation at the cytoplasmic side when ATP is not bound. This inward open form allows Kdo_2_-lipid A–core-OS entry. Stable Kdo_2_-lipid A–core-OS binding aligns TMD for ATP binding and restricts the opening of TMD. Upon ATP binding, the NBD domain intertwines, and Kdo_2_-lipid A–core-OS moves toward the periplasmic side. This coordinated movement of MsbA conformational change and LPS translocation leads to ATP hydrolysis, thus restoring the ground state inward for open confirmation of MsbA. This “outward only” mechanism for Kdo_2_-lipid A–core-OS export across the inner membrane is established based on different conformations of MsbA, derived from different Gram-negative bacterial species (Doerrler et al., 2004; Arai et al., 2017; Mi et al., 2017; Ford and Beis, 2019). Upon transport to the periplasmic region, Kdo_2_-lipid A–core-OS can undergo environmentally regulated modifications.

#### O-PS Biosynthesis and Transportation Machinery

*Klebsiella* spp. O-PS biosynthesis takes place separately at the cytoplasmic end of the inner bacterial membrane. The O-PS has four conceptually different regions: primer, adaptor, repeating unit, and terminal modification domains (Raetz and Whitfield, 2002). In general, *Klebsiella* spp. have two to five sugars in the O-PS repeating unit that are highly variable for different O-antigens (Clarke et al., 2018). The O-PS repeating unit is assembled on a lipid carrier undecaprenyl phosphate (embedded in the inner membrane) with the help of several glycosyltransferases encoded by *wecA, gmlABC, wbbMNO, wbmV, wbmW*, and *wbmX* genes and is transported to the periplasm with the help of an ABC transporter (Clarke et al., 2018). O-PS biosynthesis requires a polyisoprenoid derivative, namely, C_55_-undecaprenol phosphate (Und-P), which serves as an acceptor for O-PS chain assembly. The reaction begins with the transfer of N-acetylglucosamine (GlcNAc)-1-phosphate onto Und-P. This reaction is facilitated by GlcNAc-1-phosphate transferase (WecA) and produces Und-PP-GlcNAc, which is the primer region of O-PS. The O-PS is extended on Und-PP-GlcNAc with the help of glycosyltransferases, depending on the sugar composition of the O-PS (Meier-Dieter et al., 1992; Rick et al., 1994; Clarke et al., 1995; Guan et al., 2001; Kos et al., 2009). Depending on the O-antigen, the O-PS biosynthesis *rfb* gene cluster has 6 to 13 genes that are required for O-PS synthesis, of which 6 are essential genes (Clarke and Whitfield, 1992; Clarke et al., 2018). The 5′ end of the gene cluster has genes that encode for ABC transporters, and the 3′ end of the cluster has genes that produce glycosyltransferases. The adaptor domain, which occurs only once in an O-PS chain and acts as the connection between Und-PP-GlcNAc and the repeat unit domain, is subsequently attached to the growing O-PS. The O-PS chain extension takes place by the addition of a repeat-unit domain. The growth of O-PS occurs at the non-reducing end of the polysaccharide chain. Finally, the O-antigen length is regulated either through a covalent modification at the terminal residue of the O-PS (terminal capping/modification) or as a result of the stoichiometry of the Wzm-Wzt ABC transporter that transfers the Und-PP linked O-PS to the periplasmic end (see below).

#### O-antigen Transport Through the Wzm/Wzt System

After polymerization, the O-antigens are transported to the periplasmic-leaflet of the inner membrane by an ABC transporter that has two transmembrane domains (TMDs) (named Wzm) and two nucleotide-binding domains (NBDs) (named Wzt) (Kos et al., 2009). This O-antigen ABC transporter system is common in most of the Gram-negative bacteria. Intriguingly, in some of the *Klebsiella* spp., the O-antigen ABC transporter has an additional carbohydrate-binding domain (CBD) that is fused to the C-terminus of the NBD (Cuthbertson et al., 2005, 2007; Liston et al., 2017). Chemical modifications, such as the addition of a phosphate or methyl group at the non-reducing end of some O-antigens, provide the biosynthesis completion signal, which is recognized by the CBD to accomplish the transport (Liston et al., 2017). O12 is one such antigen that has the CBD, while such a mechanism is absent in the uncapped O-antigen biosynthesis in Kp (Bi et al., 2018).

Although structural information pertaining to the *Klebsiella* spp. Wzm/Wzt ABC transporter is unavailable, its homologous structure from *Aquifex aeolicus* has provided insights into the mechanism of O-antigen transport. Wzm/Wzt structures determined from *A. aeolicus* in ATP-free (Bi et al., 2018) and ATP-bound (Caffalette et al., 2019) forms reveal that the formation of a continuous inner transmembrane (TM) channel is wide enough to accommodate an O-antigen chain in the nucleotide-unbound conformation. ATP is seen in the bound conformation at the conserved Walker A, Walker B, and H-loop signature motifs of NDB (Davidson et al., 2008; Locher, 2016). These motifs are conserved between *Klebsiella* spp. and *A. aeolicus* and are essential for the transport of O-antigens across the inner membrane. In the complex form, the NBD adopts a compact structure and interacts with the Wzm dimer. The O-antigen chain bound to the Wzm/Wzt transporter is passed through the TM channel to reach the periplasmic face of the inner membrane, following which the lipid portion of the Und-PP-*N*-acetamido sugar moiety is inserted into the inner-membrane periplasmic leaflet (onto which the O-antigen is anchored) (Figure 2, left).

#### LPS Maturation in the Periplasm

The LPS intermediates (Und-PP-linked O-PS and Kdo_2_-lipid A–core-OS) that are transported to the periplasm are ligated with the help of WaaL ligase (a product of the *waa* gene cluster) (Regue et al., 2005). The Und-PP-linked O-PS is transferred to Kdo_2_-lipid A–core-OS by the formation of a glycosidic bond between the first sugar of the O-PS and the sugar in the outer core.

#### LPS Transport to the Outer Membrane Through LptA-G

The LPS is transported to the outer membrane through a transport system comprising seven proteins, namely, LptABCDEFG (LptA–G) (Sperandeo et al., 2007; Ruiz et al., 2008; Freinkman et al., 2011, 2012; Villa et al., 2013). All seven of the protein structures of the LPS transport system have been fully characterized (Botos et al., 2016; Dong et al., 2017; Vetterli et al., 2018; Li et al., 2019; Owens et al., 2019). Among these proteins, the LptDE and LptB_2_FG complex structures are known for *Klebsiella* spp. (Table 3), while structural information for the remaining components is available for other Gram-negative bacterial species (Vetterli et al., 2018; Li et al., 2019; Owens et al., 2019). This structural information, combined with existing knowledge of the associated transport mechanisms, has been used here to explain LPS transport in *Klebsiella* spp. Strikingly, the portal for transport of LPS molecules is formed by LptD and LptE, which is connected to a pump-like system formed by the LptB_2_FG ABC-transporter through a bridge-like structure consisting of LptA and LptC (Bishop, 2019; Li et al., 2019; Owens et al., 2019). The individual sections of this integrated LPS transporter are discussed below. As the LptA–G transporter is distributed across the inner membrane, periplasmic region, and outer membrane, this nano-machine represents a promising antimicrobial target.

#### Insertion and Translocation of LPS Into LptB_2_FG

LPS is driven across the ABC transporter LptB_2_FG (Okuda et al., 2012; Sherman et al., 2014) in a continuous flow from the periplasmic leaflet of the inner membrane to the periplasmic domain of LptC and through the transmembrane helix of LptC (Sperandeo et al., 2008, 2011; Narita and Tokuda, 2009). This is accomplished by utilizing energy derived from the ATP-hydrolysis activity of LptB (Narita and Tokuda, 2009; Sherman et al., 2014). The LptB_2_FG complex contains two transmembrane domains (LptF and LptG) and two nucleotide-binding domains (LptB_2_) (Ruiz et al., 2008; Narita and Tokuda, 2009). Both LptF and LptG contain a periplasmic β-jelly roll domain that is unique to this ABC transporter (LptB_2_FG). LPS passes into LptFG through a lateral opening formed by transmembrane helix 1 (TM1) of LptF and TM5 of LptG through an electrostatic gating mechanism (Dong et al., 2017). The LPS subsequently travels to the periplasmic domain helix (locked in-between TM1 of LptG and TM5 of LptF) of LptC (Okuda et al., 2016) in a stepwise manner (Owens et al., 2019). The soluble periplasmic protein LptA bridges LptC and the N-terminal domain of outer-membrane protein LptD by forming a head-to-tail oligomer (Suits et al., 2008) with a continuous hydrophobic groove (Bowyer et al., 2011; Sperandeo et al., 2011; Grabowicz et al., 2013; Villa et al., 2013). LptA shares a β-jelly roll fold with the periplasmic domain of LptC (Tran et al., 2010) and the N-terminal domain of LptD (Qiao et al., 2014). Strikingly, a β-jelly roll fold arrangement with a similar hydrophobic groove has also been observed in the periplasmic domain of LptF (Dong et al., 2017; Li et al., 2019; Owens et al., 2019), which could explain the transport of LPS to the outer membrane of the bacteria (as mediated by the LptFG complex).

#### LPS Assembly Onto the Outer Leaflet of the Outer Membrane

The N-terminal domain of the outer-membrane LptD is thought to be very flexible in order to maintain the physical connection and integrity of the LptCAD scaffold (Botos et al., 2016). Soon after the N-terminal domain of LptD accepts the LPS from the periplasmic protein LptA, it undergoes a significant conformational change in such a way as to open up a luminal gate formed by two periplasmic loops of LptE with LptD. The opening of the LptDE lateral gate facilitates LPS transit through the periplasmic hydrophobic groove to the extracellular region (Botos et al., 2016). Subsequent to this, the lipid A section of LPS is inserted directly into the membrane and facilitates the transition of the polysaccharide fragment through the barrel lumen to the extracellular space (Gu et al., 2015; Botos et al., 2016; Dong et al., 2017).

## A Therapeutic Perspective for Combating *Klebsiella* spp. Infections

Although antibiotics such as third-generation cephalosporins, aminoglycosides, fluoroquinolones, and carbapenems (Navon-Venezia et al., 2017; The European Antimicrobial Resistance Surveillance Network, 2018) have contributed dramatically to the reduction of morbidity and mortality associated with *Klebsiella* spp. infections, the continued emergence of cKp strains with extreme drug resistance and the newly emerged multidrug-resistant hypervirulent *Klebsiella* strains (Gu et al., 2018) limit current treatment options to eradicate infections (Brisse et al., 2009; Magiorakos et al., 2012; Doorduijn et al., 2016; Navon-Venezia et al., 2017; Martin and Bachman, 2018). Alarmingly, recent evidence suggests that *Klebsiella* has also evolved mechanisms to actively suppress innate immune responses (Bengoechea and Sa Pessoa, 2019), in addition to other well-known stealthy *Klebsiella* immune evasion strategies. Although many virulence factors are thought to be involved in the counteraction of host defenses by *Klebsiella*, only a few of these are well-studied, including CPS, LPS, fimbriae, and siderophores (Paczosa and Mecsas, 2016). As CPS and LPS actively participate in hijacking host defenses to establish infection, targeting these can prevent the growth of *Klebsiella* spp. (rather than killing the pathogen) by imposing less intense selective pressure. Ultimately, this may limit the evolution of resistant strains. Here, the biosynthesis and export of these surface-associated polysaccharides are discussed from the perspective of the treatment of *Klebsiella* infections.

CPS and LPS protect *Klebsiella* spp. from the action of complement cascade and antimicrobial peptides, as well as from engulfment and phagocytosis by host immune cells (Alvarez et al., 2000; Regueiro et al., 2006; Pan et al., 2011). In addition, CPS acts as a physical barrier to protect LPS (Merino et al., 1992; Alvarez et al., 2000). EPS (another surface-associated polysaccharide) is a component of biofilm and has been shown to interfere with the action of antimicrobial peptides of the host immune system (Bellich et al., 2018). Thus, inhibition of the LPS, CPS, and EPS biosynthesis and surface expression would be an effective approach to counteract *Klebsiella* anti-immune strategies. As the EPS secretion pathway and its structural composition in *Klebsiella* spp. is not well-understood, this review discusses the treatment strategies of *Klebsiella* infections from the perspective of CPS and LPS.

CPS and LPS are biosynthesized in cytoplasmic/periplasmic regions of the inner bacterial membrane and are transported to the bacterial surface with the help of sophisticated proteinaceous nano-machines. Thus, blocking the biosynthesis of CPS and LPS or disrupting the assembly of these nano-machineries can block the surface expression of these molecules that offer protection from the host immune response. For instance, LPS biosynthesis can be targeted in three different stages: lipid A, core-OS, and O-PS biosynthesis. Targeting the components of biosynthesis may prevent the formation of LPS and render *Klebsiella* vulnerable to host defenses. A possibility for novel antibiotic development could involve targeting the lipid A synthesizing enzymes (synthesized by the *lpx* locus), as there are no human homologs for them (Whitfield and Trent, 2014). Indeed, a recent study drawing on multi-omics data from sources including genomics, transcriptomics, structuromic, and metabolic information has listed LpxA, LpxB, LpxC, and LpxD as prioritized non-host homologous protein targets (Ramos et al., 2018). Targeting the LPS export pathway proteins represents yet another strategy. Specifically, the outer-membrane proteins [LptD and LptE (Figure 2)] involved in LPS export represent potential antibiotics targets, given that they are easily accessible (Srinivas et al., 2010; Robinson, 2019). Producing antibodies against these outer-membrane proteins is also of particular clinical interest (Storek et al., 2019). Similarly, Wzm, Wzt, and MsbA could also be targets for the development of novel antimicrobials (Alexander et al., 2018; Ho et al., 2018).

**Figure 1:**
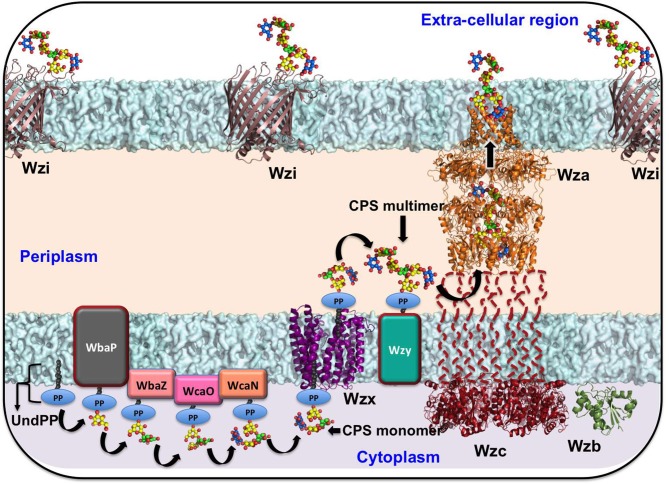
Schematic representation of *Klebsiella* spp. CPS biosynthesis and surface export machinery. The sugar precursors biosynthesized in the cytoplasm are subsequently assembled in the cytoplasmic face of the inner membrane to form the repeating unit with the help of sugar-specific glycosyl transferases WbaP (or WcaJ), followed by WbaZ, WcaN, WcaJ, and WcaO. The recognition of the CPS repeating unit by the first sugar linked to undecaprenol-pyrophosphate (Und-PP) by Wzx (a flippase) facilitates the flipping of the repeating unit to the periplasmic side. Subsequent to this event, Wzy (a copolymerase) polymerizes the repeating units. Finally, Wza (an outer-membrane translocon), Wzc (a tyrosin autokinase), and Wzb (a phosphatase) synergistically transport CPS onto the bacterial surface and anchor the CPS onto the outer-membrane protein Wzi (a lecto-aqua-porin). As structural information on the representative proteins from Kp is unknown, the structures of Wzi, Wza, Wzb, Wzc (cytoplasmic domain), and Wzx have been modeled from available reference structures through the SWISS-MODEL server (Schwede et al., 2003). *Klebsiella pneumoniae* (K20) accession numbers corresponding to Wzi, Wza, Wzb, Wzc, and Wzx are BAF47011.1, BAF47012.1, BAF4703.1, BAF47029.1, and BAT24471.1, respectively. The corresponding PDB IDs used as templates in the modeling are 2YNK (99.78%), 2J58 (99.44%), 2WMY (99.32%), 3LA6 (57.93%), and 3MKU (14.11%), respectively. The sequence identity between the query and template is indicated in the bracket.

Targeting the proteins that participate in Wzx/Wzy-dependent CPS transport and the surface expression pathway (Figure 1) may interfere with CPS export to the bacterial surface (Sachdeva et al., 2017). For instance, manipulating the function of the aqua-lecto-porin Wzi (Bushell et al., 2013; Sachdeva et al., 2016), as well as capping the extracellular side of Wza (Dong et al., 2006) involved in CPS surface expression through a novel antibiotic, would be potential targets similar to that for *E. coli*. It is worth noting that a similar strategy has been successfully demonstrated in *E. coli* Wza (Kong et al., 2013; Sachdeva et al., 2017). However, the sequence diversity of the surface-exposed region of Wza across various K-types may present a challenge in designing a common antibiotic (boxed region in Figure 3A). In contrast, Wzi is highly conserved and is a potential target for all *Klebsiella* spp. (Figure 3B).

**Figure 2:**
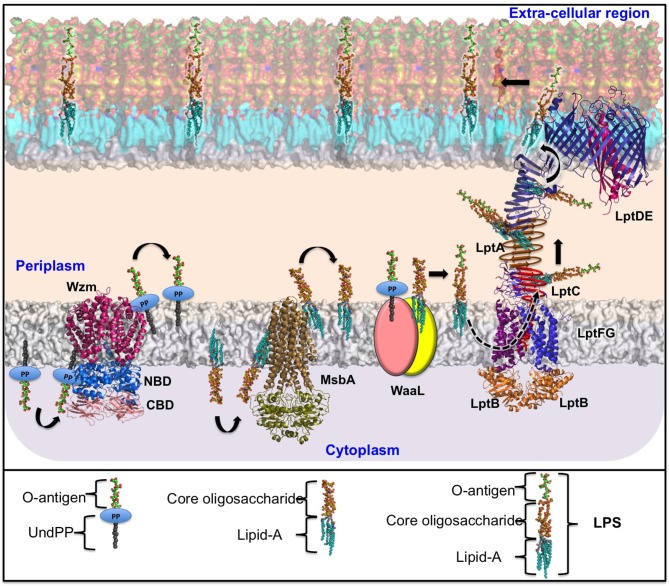
Schematic representation of *Klebsiella* spp. ABC transporter-dependent LPS assembly and transport. The O-antigen repeating unit is synthesized in the cytosol with the help of the corresponding glycosyltransferases and is subsequently polymerized by Wzy and transferred to the periplasm by an ABC transporter (Wzm/Wzt complex). In a similar fashion, the Kdo_2_-lipid A–core oligosaccharide biosynthesized in the cytoplasmic region is flipped to the periplasmic region through the ATP-driven MsbA. Following this, the matured O-polysaccharide and lipid A-core-oligosaccharide are ligated by WaaL ligase in the periplasmic region. The completely grown LPS is transported to the bacterial surface through LptA-G assembly as indicated. For the purpose of illustration, the LptB2FG (PDB ID: 5L75) and LptDE (PDB ID: 5IV9) structures are taken directly from Kp, while the Wzm/Wzt complex and MsbA proteins are homology-modeled using structures available in other organisms as templates. The reference PDB IDs for Wzm, Wzt-NBD, and Wzt-CBD are 6AN7 (34.5%), 6AN5 (46.32%), and 2R5O (100%), respectively. The sequence identity between the template and the Kp are given in brackets. The NCBI accession numbers corresponding to the Kp protein sequences are CZQ25306.1 (Wzm) and CZQ25307.1 (Wzt-NBD and Wzt-CBD). LptA and LptC are indicated by schematic representation. The helical and beta-jelly conformation of LptC is shown in red. The beta-jelly conformation of LptA is colored dark gold. As WaaL structural information for any Gram-negative organism is unavailable, the two domains of WaaL are represented in yellow and peach-colored ovals. Note that for the purpose of illustration, O3 has been considered as a case in point. Individual parts of the LPS and O-antigen are annotated separately at the bottom of the figure.

**Figure 3:**
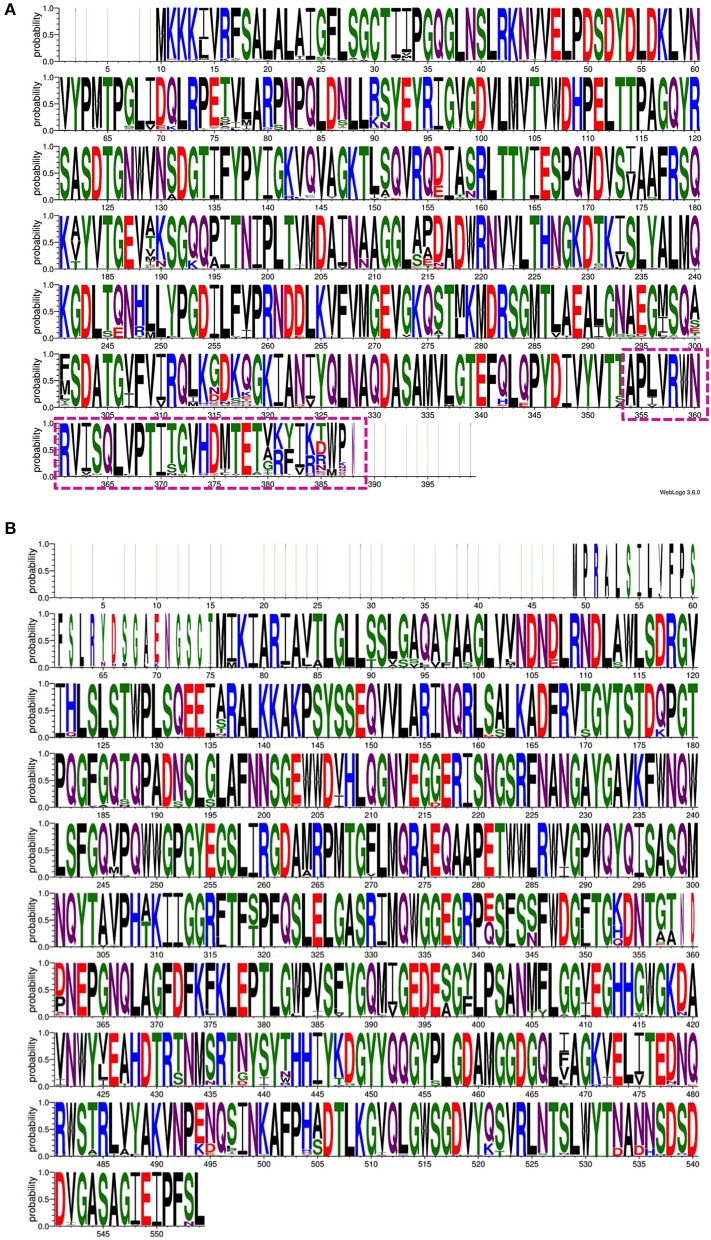
An amino acid sequence logo constructed using the multiple sequence alignment of 139 Wza protein sequences **(A)** and 138 Wzi protein sequences **(B)**. Note the variation observed in the C-terminal region (transmembrane region) of Wza that faces the extracellular region of the bacterial cell (dash-box) **(A)**. In contrast, Wzi sequences are highly conserved between different serotypes of *Klebsiella* spp. Note that non-redundant sequences that have a defined K-type are used to generate sequence logo.

Another approach for the treatment of *Klebsiella* infection involves the development of antibodies targeting CPS and LPS (Szijarto et al., 2017; Diago-Navarro et al., 2018; Kobayashi et al., 2018). Cell surface carbohydrate-based vaccines (Hutter and Lepenies, 2015) can be an effective choice for combating *Klebsiella* infections (Cryz et al., 1985; Cross, 2014; Seeberger et al., 2017; Adamo and Margarit, 2018; Hegerle et al., 2018; Micoli et al., 2018). Glycan epitopes, namely, the antibody-interacting and minimal antigenic determinant of O- or K- antigens, can be used in vaccine development. The heterogeneity and complexity of O- and K-antigens of different *Klebsiella* serotypes may pose a challenge to the development of a polyvalent vaccine against all *Klebsiella* infections. Fortunately, only a few O- and K-antigens are found in clinical isolates; thus, they can be used in the development of a novel immunogenic polyvalent glycoconjugate *Klebsiella* vaccine with the help of improved vaccine technology. Multiple interactions between protein and glycan is essential at different stages of the immune response. Identification of surface saccharide epitope patterns in clinical/hypervirulent strains and their use in the design of a unique synthetic glycan epitope conjugated with an immunogenic carrier protein may be useful in the development of an effective multivalent glycoconjugate *Klebsiella* vaccine.

Although *Klebsiella* spp. have 12 O-antigens, seroepidemiological investigations have revealed only four *Klebsiella* O serotypes found in clinical isolates (Edelman et al., 1994; Cryz et al., 1995; Trautmann et al., 2004). Thus, *Klebsiella* anti-endotoxin vaccines/antibodies can be developed based on the O-antigen structure of clinical isolates of *Klebsiella* spp. Protection against Kp through anti-LPS antibodies has been successfully demonstrated (Cohen et al., 2017; Pennini et al., 2017; Hegerle et al., 2018). Although thermostable LPS is a strong immune activator, Kp quite often uses modifications of lipid A of LPS in such a way that it is no longer recognized by certain immune receptors such as TLR4 (Llobet et al., 2015). This helps it evade the complement system and to survive within the host during colonization and infection (Llobet et al., 2011, 2015; Kidd et al., 2017; Mills et al., 2017). Modification of the polysaccharide composition of the O-antigen side chain (which is exposed to antibodies) and elongation of the O-antigen has also been documented (Doorduijn et al., 2016). Kp strains with a long O-antigen produce a high-molecular-weight (smooth phenotype) LPS that is less susceptible to serum killing, as compared to strains lacking an O-antigen side chain with a low-molecular-weight (rough phenotype) LPS (Ciurana and Tomas, 1987; Mccallum et al., 1989). For example, D-galactan I to D-galactan III structure modification of the O-antigen is found to improve Kp survival in human serum compared to strains expressing D-galactan I (Szijarto et al., 2016). Similarly, an epidemic multidrug-resistant Kp clone (Tzouvelekis et al., 2013) was found to have a modified O-antigen structure (Wyres et al., 2015; Szijarto et al., 2016). Modification of the glycan structures at the terminal end of the O-antigen has also been shown to alter complement activation in Kp (Tytgat and Lebeer, 2014; Adamo and Margarit, 2018).

CPS could also be exploited to counteract *Klebsiella* anti-immune strategies. Recognition of this possibility has led to the development of a 24-valent CPS-based vaccine for *Klebsiella* (Cryz et al., 1991; Edelman et al., 1994; Campbell et al., 1996; Donta et al., 1996). Although a phase 1 trial of the vaccine has shown it to be immunogenic and non-toxic (Edelman et al., 1994), no further developments have been reported in the last two decades. Similar to LPS, the capsule also undergoes modifications to resist the host complement system (Wyres et al., 2015; Szijarto et al., 2016). This may pose a challenge in developing a vaccine against *Klebsiella* spp. infections. Chemical modifications in K-antigen structures, such as acetylation and deacetylation (Hsu et al., 2016), may also bring about differential effects in CPS antigenicity, representing yet another challenge in the development of vaccines against *Klebsiella* spp. K2-antigen-lacking mannobiose or rhamnobiose produced by a Kp strain escapes host recognition during the host innate immune response (Sahly et al., 2009). It is worth noting that hvKp strains are frequently found to have K2 antigens.

Use of exogenous cholesterol and bacteriophage depolymerase against *Klebsiella* infections represents yet another promising approach. It has been shown that exogenous cholesterol increases macrophage-mediated phagocytosis by down-regulating the expression of genes responsible for LPS core oligosaccharides production, as well as reducing the anti-phagocytic properties of the Kp capsule (Ares et al., 2019). The discovery that bacteriophage capsule depolymerases can be used against *Klebsiella* capsule types KN1, KN3, KN4, and K56 represents a potential approach for the treatment of Kp infections (Pan et al., 2019).

Significant progress has been made in understanding *Klebsiella* immune evasion strategies. As the CPS and LPS of *Klebsiella* spp. play an important role in hijacking host defenses, targeting these virulence factors may be an efficient strategy against *Klebsiella* infections. The known structural components of *Klebsiella* CPS and LPS export machineries could be useful in the design of novel antibiotics. However, heterogeneity in sugar composition, glycosidic linkage, stereoisomeric forms, and the concomitant variation in the proteins involved in biosynthesis and transport may pose a challenge in the design of antibiotics and vaccines that can be used against diverse *Klebsiella* spp. In addition, the ability of *Klebsiella* spp. to modify components of the CPS and LPS may be another concern. Recent developments in gene sequencing techniques in combination with a metagenomic approach to the investigation of Kp clinical strains help in the design of polyvalent vaccines. A combinatorial therapy involving *Klebsiella* vaccines against surface polysaccharides and antibiotics inhibiting surface antigen assembly may represent the most promising approach.

## Author Contributions

TR designed and supervised the entire project. LP and TR wrote the manuscript.

### Conflict of Interest

The authors declare that the research was conducted in the absence of any commercial or financial relationships that could be construed as a potential conflict of interest.
